# Modulatory Effects of Breed, Feeding Status, and Diet on Adipogenic, Lipogenic, and Lipolytic Gene Expression in Growing Iberian and Duroc Pigs

**DOI:** 10.3390/ijms19010022

**Published:** 2017-12-22

**Authors:** Rita Benítez, Almudena Fernández, Beatriz Isabel, Yolanda Núñez, Eduardo De Mercado, Emilio Gómez-Izquierdo, Juan García-Casco, Clemente López-Bote, Cristina Óvilo

**Affiliations:** 1 Department of Animal Breeding, INIA, Ctra. De la Coruña, km 7.5, 28040 Madrid, Spain; rmbenitez@inia.es (R.B.); afedez@inia.es (A.F.); nunez.yolanda@inia.es (Y.N.); garcia.juan@inia.es (J.G.-C.); 2 Faculty of Veterinary, Universidad Complutense de Madrid, 28040 Madrid, Spain; bisabelr@pdi.ucm.es (B.I.); clemente@vet.ucm.es (C.L.-B.); 3 Pig Test Center ITACYL, Hontalbilla, 40353 Segovia, Spain; ita-merpened@itacyl.es (E.D.M.); gomizqem@itacyl.es (E.G.-I.)

**Keywords:** nutrigenomics, diet, fasting, adipose tissue, lipid metabolism, gene expression and Iberian pig

## Abstract

Meat quality depends on tissue composition which is in turn influenced by different factors, such as diet, genotype, age, or sex. We evaluated the effects of breed, 24 h fasting, and dietary energy source (HO: oleic acid versus CH: carbohydrates) on the expression of candidate genes involved in adipogenesis, lipogenesis, and lipolysis in the adipose tissue from Iberian and Duroc growing pigs. The Iberian pigs showed greater feed intake, backfat thickness, and saturated fatty acids (SFA) content in the subcutaneous fat, whereas the Duroc pigs had greater ham weight and polyunsaturated fatty acids (PUFA) content. In both breeds, the diet induced changes in the fatty acid (FA) composition of subcutaneous fat samples. The HO group had higher monounsaturated fatty acids (MUFA) and oleic acid, and lower SFA than the CH group. Regarding gene expression, breed and feeding status (fasting versus postprandial) had significant effects on gene expression, with quantitative interactions between them, while diet showed negligible effects. In general, adipogenic and lipogenic genes were upregulated in the Iberian pigs and in postprandial samples. In contrast, the expression of lipolytic genes showed complex interaction effects. Our results agree with the phenotypic differences between the Iberian and Duroc breeds and with the inhibition of lipogenesis by fasting. Quantitative interactions between breed and feeding status effects were observed, which indicates a different response to fasting of the two breeds, with the obese Iberian breed showing a more stable expression of lipogenic genes. These results highlight the complexity of lipid metabolism regulation, especially in relation to lipolysis processes.

## 1. Introduction

The Iberian pig is the main representative of the Mediterranean traditional fatty pig breeds. This breed has an outstanding commercial and socioeconomic value because of the employment of natural resources in its extensive production system and because it is the basis for the production of high quality dry-cured meat products [1]. The Iberian breed is characterized by a great appetite, fatness, and protein turnover ratio resulting in low lean growth efficiency [2], and is traditionally fed according to a feeding system based on the intake of acorns and on pasture, in the final fattening period. This fattening system is a reference model for the sustainable production of many local Mediterranean breeds [3]. The Iberian pig production is based on both purebred Iberian and crossbred Duroc × Iberian pigs. These two genotypes show important phenotypic differences in growth, fattening, tissue composition, muscle differentiation, and several metabolic processes from early developmental stages [2,4]. Specifically, purebred Iberian animals are characterized by a lower lean growth efficiency and a higher meat quality than the Duroc genotypes, although a precise characterization of the phenotypic differences between the pure breeds has not been reported yet.

Fat content and composition largely determine meat quality [5] and are influenced by individual factors, including genetic background, age, and sex, and also by environmental factors such as nutritional composition. Diet is a main factor influencing animal body and tissue composition and may be employed to improve the nutritional value of the meat [6]. Diet components can be directly stored in the animal tissues and they may also affect metabolism by influencing gene expression. Monounsaturated fatty acids (MUFA)-enriched diets with the inclusion of high oleic acid sunflower oil, are being used in Iberian pig intensive fattening systems in order to increase oleic acid and to be used as an alternative to the traditional fattening system of *Montanera* [7,8]. Recently, the impact of high oleic sunflower oil (HO)- versus carbohydrate (CH)-enriched diets on tissue composition and their short- and long-term effects on gene transcription have been evaluated in pure Iberian pigs [9,10,11,12]. In previous works, the effects of diet composition on gene expression were small and conditioned by several factors, such as timing and feeding status. Moreover, the diet effects on metabolism may depend on the peculiar breed and genotype employed. Specifically, the high basal lipogenesis that is characteristic of the Iberian breed tissues may be associated with a lower responsiveness to dietary interventions regarding lipid metabolism. 

Also, in our previous work performed with Iberian pigs, [10] the effects of a period of 18 h fasting on lipogenic gene expression was tested. This medium-term fasting only downregulated the expression of the *PPARG* gene among a panel of lipogenesis candidates. The scarce transcriptional response to fasting was unexpected and could be related to the duration of the fasting applied, as longer fasting periods are expected to induce a higher response in gene expression. On the other hand, these scarce effects of fasting could also be a consequence of the genetic type employed (pure Iberian animals), in which the high lipogenesis potential may limit or delay the transcriptional response to the lack of energy supply. Understanding the molecular mechanisms driving the high potential for fat deposition in Iberian pigs in comparison to other breeds, even in adverse situations of caloric restriction, would have a relevant scientific interest from an animal genetics and production perspective. Moreover, it also could provide relevant data to deepen the knowledge of these complex processes in other obese animals including humans, as the Iberian pig is considered an adequate animal model for obesity and metabolic alterations [13].

Fat deposition is the result of the balance between the processes of lipogenesis and lipolysis [14]. Previous nutrigenomic works addressed the study of lipogenesis at the transcriptional level, following diet and fasting treatments [9,10]. On the other hand, previous transcriptome studies compared muscle gene expression of Iberian versus crossbred animals [2,15]. These studies suggested a more active lipogenesis in pure Iberian animals, but also showed that some lipolytic genes were differentially expressed in the two genotypes, and indicated the relative contribution of both processes in the final differences in fat deposition [15], highlighting the interest of studying both processes simultaneously.

Thus, in order to better understand the effects of diet and fasting on gene transcription and lipid metabolism, and the potential differential response between breeds, the Iberian and another reference lean breed must be jointly studied in the same experimental conditions, using both a dietary intervention and a longer fasting period. In agreement with previous considerations, this study was conducted to investigate the effects of breed (Iberian vs. Duroc), a long period of fasting (24 h), and dietary energy source (HO vs. CH) on adipose tissue composition and gene expression of candidate genes involved in adipogenesis, lipogenesis, and lipolysis in growing pigs.

## 2. Results

Growth, fattening, and fatty acid (FA) composition data corresponding to the pig samples obtained at slaughter were employed to analyze the breed and diet effects. Adipose tissue samples obtained by biopsy four days before slaughter were employed to study the effects of breed, diet, and 24 h fasting on gene expression.

### 2.1. Diet and Breed Effects on Phenotype and Animal Tissue Composition

The two dietary groups (HO, CH) showed similar body weight, backfat thickness, and ham weight at the end of the experiment. Regarding the breed effects, a higher backfat thickness (24.1 mm versus 10.7 mm in loin and 27.8 mm versus 15.7 mm in ham, *p* < 0.001) and a higher average feed intake (2 kg versus 1.7 kg, *p* < 0.05) were registered in the Iberian pigs, whereas a higher ham weight (4.5 kg versus 3.5 kg, *p* < 0.001) was registered in the Duroc pigs.

The FA profile was studied in two different tissues: subcutaneous backfat and subcutaneous ham fat (inner and outer layers). The behavior of both layers was very similar at each location, thus the results of the diet and breed effects are presented and discussed for the inner layer (Table 1 and Table 2). In both locations (backfat and ham fat), FA composition showed significant differences between dietary groups and breeds. The main diet effects were observed for saturated fatty acids (SFA) and MUFA contents. The HO group showed a higher MUFA content, and the CH group showed a higher SFA content. These differences were mainly due to higher palmitic (C16:0) and stearic (C18:0) acids in the CH, and higher palmitoleic (C16:1) and oleic (C18:1) in the HO group. The n-6/n-3 ratio was slightly higher in the CH diet in both locations. 

In the backfat, the main breed effects were observed for SFA, MUFA, and PUFA contents, with higher SFA in the Iberian pigs and higher MUFA and PUFA in the Duroc pigs. These changes were mainly due to a major increase of palmitic and stearic acids in the Iberian pigs and to an increase of palmitoleic, linoleic, linolenic, and eicosadienoic fatty acids in the Duroc pigs, although some other minority of FA was also affected. No significant difference in oleic acid content was observed between the breeds.

Similarly, in ham fat, the main breed effects were observed for SFA and PUFA contents, with higher SFA in the Iberian pigs and higher PUFA in the Duroc pigs. These effects were a consequence of higher palmitic acid in the Iberian pigs and higher linoleic and linolenic acids in the Duroc pigs. The n-6/n-3 ratio was higher in the Duroc pigs in both adipose depots. This change was in agreement with higher levels of linoleic acid in the Duroc pigs, which is the main representative of n-6 FA and the precursor of the synthesis of arachidonic acid (C20:4n-6).

Only two significant diet x breed interactions were observed in the subcutaneous backfat for eicosadienoic (C20:2) and eicosatrienoic (C20:3n-3) fatty acids, with a higher amount of these FA in the Duroc pigs fed a CH diet than in the rest of the groups.

### 2.2. Breed, Feeding Status, and Diet Effects on the Expression of Candidate Genes 

Thirteen selected candidate genes were successfully quantified in mRNA samples from ham subcutaneous adipose biopsies obtained in vivo after 24 h of fasting and 3 h after refeeding.

**Figure 1:**
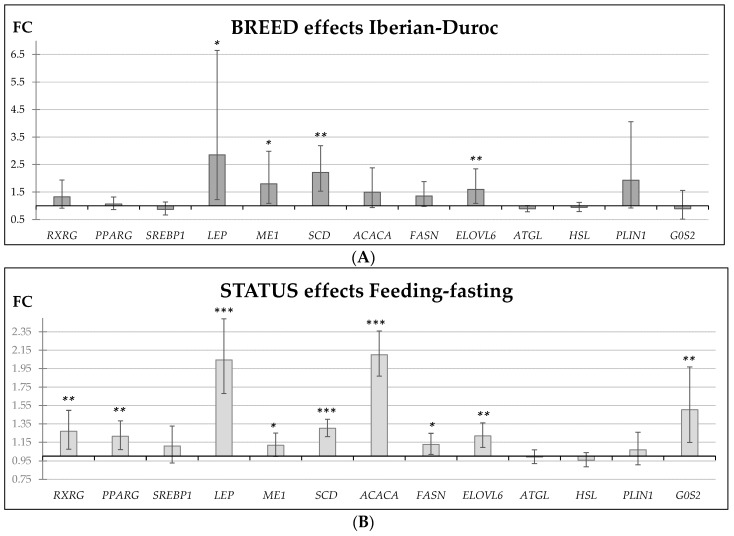
Fold-change ratios (FC) of candidate genes expression in ham subcutaneous adipose biopsies (**A**) Iberian (*n* = 29) versus Duroc (*n* = 19) (**B**) Feeding versus fasting status (*n* = 48). The error lines indicate the standard errors. FC values > 1 indicate higher expression in Iberian pigs and postprandial status. * *p* < 0.05, ** *p* < 0.001, *** *p* < 0.0001.

The effects of breed, feeding status, and diet on gene expression are shown in Table 3 and Figure 1. Breed significantly affected the expression of several analyzed genes. *LEP*, *ME1*, *SCD*, and *ELOVL6* showed higher expression in biopsies obtained from the Iberian pigs, and *FASN* and *PLIN1* showed the same trend. On the contrary, the expression of the lipolytic gene *ATGL* tended to be higher in the Duroc samples. Fold-change (FC) ratios of *SCD* and *LEP* genes were considerably high for the breed effects (Figure 1A). The FC values ranged from 2.85 for the *LEP* gene to 1.60 for the *ELOVL6* gene.

The fasting and feeding statuses significantly affected the expression of most analyzed genes. *RXRG*, *PPARG*, *LEP*, *ME1*, *SCD*, *ACACA*, *FASN*, *ELOVL6*, and *G0S2* showed higher expression in the biopsies obtained in the postprandial status. *SREBP1*, *ATGL*, *HSL*, and *PLIN1* were not affected by the feeding status. The FC ratios calculated for the *ACACA* and *LEP* genes were considerably high for the status effect (Figure 1B). FCs ranged from 2.10 for the *ACACA* gene to 1.12 for the *ME1* gene.

Diet showed negligible effects on gene expression. Only the *PLIN1* gene was upregulated in the CH group.

**Figure 2:**
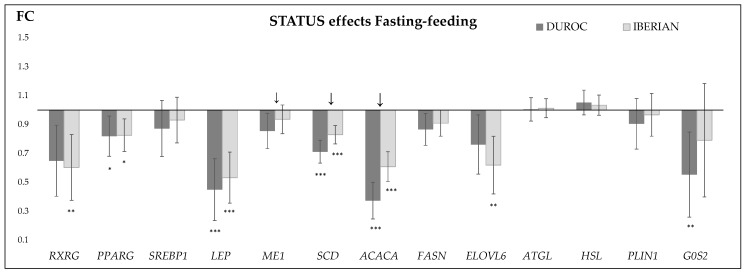
Fold-change (FC) ratios of candidate genes expression between fasting versus feeding status in ham subcutaneous adipose biopsies obtained from Duroc (*n* = 19) and Iberian (*n* = 29) pigs. The error lines indicate the standard errors. FC values > 1 indicate higher expression in fasting status. * *p* < 0.05, ** *p* < 0.01, *** *p* < 0.0001. Breed × status interaction effects are indicated with an arrow (↓) (*p* < 0.07, *p* < 0.03 and *p* < 0.0001 for *ME1*, *SCD*, and *ACACA* genes, respectively).

The results corresponding to the interaction effects are shown in Table 4 and in Figure 2, Figures S1 and S2. Significant breed × status interaction effects were found for the *SCD* and *ACACA* genes (*p* < 0.03 and *p* < 0.0001, respectively) and a similar tendency was found for *ME1* (*p* < 0.07) (Figure 2).

A significant breed × diet interaction effect was found for the *ATGL* gene (*p* < 0.006), and significant diet × status interaction effects were found for *SREBP1*, *ACACA*, *ATGL*, *HSL* and *PLIN1* (*p* < 0.03, *p* < 0.004, *p* < 0.02, *p* < 0.03, and *p* < 0.0004 respectively) (Table 4 and Figures S1 and S2).

## 3. Discussion

### 3.1. Diet and Breed Effects on Phenotype and Tissue Composition

Different works reported no effect of dietary fat source or saturation on the growth performance and carcass characteristics in pigs [16,17,18], matching our findings. In relation to the FA composition, our study shows significant effects of diet in both breeds and in the fatty acid profiles of both subcutaneous backfat and ham fat. Both depots responded in a similar way to the diet reflecting in part the composition of the diet received, with much higher MUFA in the HO group, as a result of a major increase in oleic acid, as expected. The content of SFA was higher in the CH group, although the CH diet included a lower proportion of palmitic and stearic acids, indicating de novo synthesis of FA from the available carbohydrates in this group. The PUFA content was also slightly affected by diet, and was higher in the HO group because of an increase in linoleic acid, in agreement with the diet composition. All these results are coincident with previous works [7,9] and are consistent with the deposition of dietary FA and the induction of lipogenesis by dietary CH. Different FA values and ratios are usually employed as measures of tissue metabolism and of the tissue nutritional value in relation to human health. Particularly, fat saturation and high n-6 PUFA or n-6/n-3 ratio, are related to human diseases, such as atherosclerosis, cardiovascular disease, metabolic syndrome, or cancer [19,20]. The supplementation with high oleic sunflower oil reduced the saturation of pig fats and the n-6/n-3 ratio in both breeds, which positively affects the meat nutritional quality.

Most previous works of oleic acid supplementation were performed with Iberian type pigs, and the analyses were performed in backfat samples. The present results extend the findings to another breed that was not examined before, the cosmopolite Duroc breed, in which, according to our results, the employment of an oleic acid-supplemented diet may be a useful tool to improve the sensorial, organoleptic, and nutritional quality of meat products. This finding is especially interesting in a breed which, in contrast to the Iberian, has moderate meat quality attributes. Moreover, our results were validated in subcutaneous ham fat, whose composition is essential for the production of the cured ham, the main pig cured product.

To the best of our knowledge, this is the first work in which purebred Iberian and purebred Duroc animals have been compared in identical environmental conditions. These results are relevant from a productive and scientific perspective. The main phenotypic traits related to fatness and premium cut yield (backfat thickness, ham weights, and feed intake) strongly differed between breeds, as expected. The Iberian pigs showed higher fatness and appetite, and the Duroc showed higher ham weight. The Iberian pig breed is a local fat breed characterized by an extreme adipogenic potential, rusticity, and low productive efficiency, with bad conversion rates, low meat yield, and low reproductive efficiency [1]. Also, the Iberian pigs are characterized by high levels of voluntary feed intake [21] in agreement with our findings. This high appetite is an adaptative mechanism typical of animals subjected to oscillations in food availability (thrifty genotype) [2,9] and is concomitant with higher plasma levels of leptin hormone in comparison to other breeds [22]. Thus, in the Iberian breed high levels of leptin fail to reduce appetite, fitting with a pattern of obesity by leptin resistance.

Our Iberian and Duroc pigs clearly differed in fattening and appetite, however it is interesting to note that growing Iberian and Duroc pigs differed in ham weight, whereas no difference was found in body weight. Differences in ham measures between Iberian pig genotypes were previously observed in juvenile and adult pigs [23,24]. Although many studies have reported differences in weight between these genotypes at birth and at the final slaughter age, the differences in body weight and size are not evident at the weaning [9] nor at the growing [2] periods, in concordance with the present findings. This fact may be associated with the thrifty genotype of the Iberian pigs, which might lead to high levels of voluntary feed intake and, or to low energy expenditure during suckling and early growth periods, without being reflected on adult pigs because of the lower growth potential of the mature pure Iberian animals [2].

In the comparison between breeds, significant differences were also observed for the FA composition. Jointly, higher SFA and lower PUFA were observed in the Iberian compared to the Duroc pigs. These results are in agreement with the greater de novo fat synthesis in the Iberian pig tissues, leading to a higher SFA content and to a dilution effect for other FA. On the other hand, the level of PUFA in porcine tissues is only dependent on diet. Hence, the significantly higher levels of PUFA found in the subcutaneous backfat and ham subcutaneous fat in the Duroc pigs could be also explained by a higher ability of the Duroc genotype to store dietary unsaturated lipids in their tissues [25].

### 3.2. Breed, Feeding Status, and Diet Effects on Candidate Genes Expression

The expression of a panel of adipogenic, lipogenic, and lipolytic candidate genes was explored, allowing the identification of relevant breed and fasting effects on transcription, which gives insights into the differential regulation of lipid metabolism between a local obese and a cosmopolite breed. This work was only approached at the transcriptional level, and thus the lack of activity information may be considered a limitation. However, gene expression measurements are considered reasonable measures of activity, as a close relationship has been observed between these measurements [26,27].

The breed had a considerable effect on gene expression, with genes involved in energy balance and lipogenesis being upregulated in the Iberian pigs. The leptin gene showed the highest upregulation in Iberian adipose tissue (2.85 times) in agreement with its high leptin protein levels [22], although this is the first report of the breed effect at the transcriptional level. The remaining genes upregulated in the Iberian pigs are directly involved in de novo lipogenesis, in agreement with its phenotype. The *SCD* gene also showed a strong upregulation in Iberian samples (2.21 times), matching with the findings of a previous muscle transcriptome comparison between Iberian and Duroc crossbred pigs [15]. This result is also in agreement with the segregation of a known polymorphism, AY487830:g.2228T > C in the *SCD* gene promoter [28,29]. This single nucleotide polymorphism (SNP) has an allele (g.2228T) associated with a higher expression of the gene, which is fixed in our Iberian pigs but segregating in the Duroc ones. The *SCD* gene upregulation in the Iberian pigs may be associated with the higher desaturation potential of this breed [30], although, in our work, this difference in gene expression is not translated in a higher MUFA content in Iberian pig tissues. One explanation for this fact is the predominance of de novo lipogenesis over FA desaturation in the growing period analyzed here, which may lead to intense increases in SFA and to a dilution of the other FA. We did not detect any breed effect on transcriptional regulators involved in adipocyte differentiation (*RXRG*, *PPARG*, *SREBP1*), in contrast to previous findings in younger animals [31], probably in agreement with the late moment of sampling regarding cellular differentiation processes. Jointly, the breed effects on lipogenic genes support the predisposition for obesity of the Iberian breed, provide a molecular basis for the development of leptin resistance and metabolic syndrome in the Iberian pigs fed high fat diets [13], and reinforces the usefulness of this pig as a biomedical model for obesity and metabolic disorders.

Nine out of the thirteen analyzed genes were significantly affected by the feeding status, with all differentially expressed genes being upregulated in the fed status. Specifically, differential expression was found for all the lipogenic genes (*SCD*, *ME1*, *ACACA*, *FASN*, *ELOVL6* and *LEP*), two regulators (*RXRG* and *PPARG*), and one lipolysis regulator gene (*GOS2*). Fasting, including short-term food deprivation, is known to reduce lipogenesis and induce lipolysis [32,33,34]. Our results agree with fasting inhibiting lipogenesis and adipocyte differentiation. Nevertheless, regarding lipolysis, no response was observed after 24 h of fasting for the main lipolytic genes *ATGL*, *HSL* and *PLIN1* and *GOS2* was activated after refeeding. Although this last gene is functionally involved in lipolysis, it is a negative regulator of lipolysis, and previous studies also reported its downregulation after 24 h of fasting [35].

In our previous work [10], an 18 h fasting period affected the expression of the *PPARG* gene, which was activated after feeding, but had no effect on downstream genes. The actual results confirm that this unexpected, previous result was related, at least in part, to the duration of the fasting applied, as a 24 h period has a much more intense effect on gene expression, in agreement with other works [36,37,38]. Thus, a medium-long food deprivation period (24 h of fasting) seems to be necessary to stop lipogenesis in these fatty animals. On the other hand, our previous results suggested that, in the Iberian pigs, as previously proposed in obese mice, de novo lipogenesis may persist in adipose tissue during fasting [36]. In order to test the persistence of lipogenesis in the Iberian breed during fasting, the breed and the fasting effects were considered jointly. Significant breed × feeding status interaction effects were observed for the *SCD* and *ACACA* genes, and a similar trend was observed for *ME1*. In the three cases, the interactions were quantitative and revealed that the inhibition of these genes after 24 h of fasting was more intense in the Duroc pigs, or, in other words, their expression was more stable in the Iberian pigs (Figure 2). Moreover, the expression patterns of several other lipogenic genes (*SREBP1*, *LEP*, *FASN*) were similar, with a more pronounced effect of fasting in the Duroc pigs. Although the interaction effects for some genes did not reach statistical significance, jointly, these results support a more persistent lipogenesis in the Iberian than in the Duroc breed. This finding provides novel information on the regulation of fat deposition in genetically determined obesity, which was until now based on previous scarce results derived from mice with a diet-induced obesity [36]. Moreover, our results extend the findings to a large fasting (24 h) period versus an overnight fast, and to new molecules that were not studied in the previous works, such as *SCD*, *ME1*, and *LEP*.

Surprisingly, the diet did not affect the expression of any analyzed gene but *PLIN1*, upregulated in the CH group. The scarce diet effects were unexpected, as effects of similar diets were found in the Iberian pigs in early and final growth stages [9,10]. Nevertheless, the effects of the FA dietary composition on gene expression are known to be scarce, small, and difficult to measure; in addition, these effects are deeply modulated by factors such as genotype or timing [9]. Thus, the interaction effects might have conditioned our ability to detect and understand the diet effect on metabolism. In our previous work [10], we found evidence of a significant interaction diet × status on the *ME1* gene. In our present results, qualitative interactions diet × status were observed for *ACACA*, *SREBP1*, *ATGL*, *HSL*, and *PLIN1*. Regarding the lipogenic genes *ACACA* and *SREBP1*, the diet effects on gene expression seemed to be opposite in the fasting and feeding states (Figure S2). For the lipolytic genes *ATGL*, *HSL*, and *PLIN1*, a similar qualitative interaction was found, with the same pattern for the three genes, but with an opposite trend to that of the lipogenic ones. These interactions may be related to differences in the timing and availability of nutrients in the blood after refeeding between these diets, as the CH diet is expected to increase glucose levels faster than the HO diet. Thus, for instance, the upregulation of lipogenic genes in the CH group after refeeding is consistent with the expected major postprandial increase in serum glucose in animals fed a diet rich in carbohydrates.

In addition, a significant interaction breed × diet was found for the *ATGL* gene (*p* < 0.006), and similar trends were found for the *ME1*, *HSL*, and *PLIN1* genes (*p* < 0.07 for all) (Figure S1). Although these results should be considered with caution because of the lack of statistical significance for some genes, the coincidence in the observed patterns for all genes with a functional relationship suggests an underlying biological basis that should be explored in future works. Interestingly, the three main lipolytic genes, *ATGL*, *HSL*, and *PLIN1*, present exactly the same pattern of diet × breed and diet × status interactions, in agreement with their common biological role. According to findings in mice and humans, the main lipases *ATGL* and *HSL* are downregulated in obese individuals [34,39,40], and lipolysis is assumed to be impaired in obesity [41,42,43]. In this study, we would have expected to find lower lipolytic gene expression in the Iberian breed and higher lipolytic gene expression in fasted animals. The lack of significant main effects is related to the interactions breed × diet and diet × status. For example, for the *ATGL* gene, downregulation in the Iberian pigs was only observed under the CH diet, and downregulation by fasting was only observed under the HO diet. Undoubtedly, all these complex interactions hinder our ability to detect and interpret the influence of the different factors on gene expression.

Finally, it is known that a high level of leptin induces lipolysis at the adipose tissue level [34]; in our case, the leptin gene showed the highest upregulation in the Iberian pigs (2.85 times), but we did not find a greater lipolysis in the Iberian pigs. Leptin effects are dependent on the leptin receptor, and it is known that Iberian pigs show leptin resistance, related to the presence of polymorphisms in the *LEPR* gene that either reduce its expression or reduces its signaling ability [44,45]. Thus, the Iberian pigs would be resistant to leptin-induced lipolysis.

## 4. Materials and Methods

### 4.1. Animals 

The current study was carried out at the facilities of the Pig Test Center ITACYL (Hontalbilla, Segovia, Spain). The animal manipulations were performed in compliance with the regulations of the Spanish Policy for Protection of Animals employed in Research and other scientific purposes RD53/2013, which meet the European Union Directive 2010/63/EU about the protection of animals used in experimentation. The project was approved on 20 March 2015, by the *Comunidad de Madrid* animal welfare and protection committee, with reference number PROEX-007/15. The study comprised a total of 30 Iberian and 19 Duroc males born in 19 contemporary litters. These animals were kept under identical management conditions. At 10 weeks of age (SD = 1.6 days), the animals were distributed in two experimental groups and fed two different isocaloric and isoproteic diets (3.3 kcal of digestible energy and 15.6% of crude protein) provided ad libitum and differing in the energy source: HO diet enriched with 6% high oleic sunflower oil (17 Iberian and 10 Duroc pigs) and CH standard diet with carbohydrates as an energy source (13 Iberian and 9 Duroc pigs). Fresh water was provided ad libitum. The animals started the experimental period at 19.9 kg (SD = 3.8 kg) of average live weight (LW) and were slaughtered after 47 days of treatment, with 51.2 kg (SD = 5.5 kg) of average LW. The batch feed intake was recorded daily. Feeds composition is shown in Table 5.

Four days before slaughter, ham subcutaneous fat samples were obtained in vivo by short-biopsies after a 24 h fasting period and 3 h after refeeding (postprandial sampling). Tranquilization was performed by intramuscular injection of 20 mg of azaperon per 10 kg live weight (Stresnil; Esteve, Barcelona, Spain) 1 h before microbiopsies were taken. A cylindrical biopsy device with a diameter of 5 mm was employed for biopsies, which were taken under local anesthesia with 2% lidocaine-HCL (Alphacaine; Fendigo, Brussels, Belgium). After the procedure, the zone was treated with oxytretracycline and lidocaine (Veterin Tenicol; Lab. Intervet S.A., Salamanca, Spain). The pigs did not suffer any pain because of the analgesia employed. Similar procedures have been used before [6,24]. The biopsy samples were placed in cryotubes, snap frozen in liquid nitrogen, and stored at −80 °C until gene expression analyses.

Slaughter was performed at an experimental slaughterhouse (Hontalbilla, Segovia, Spain) after an overnight fasting period. Subcutaneous fat samples were collected from the carcasses at two levels: backfat at the last rib and ham fat. The fat was then separated into outer and inner layers, which were separately analyzed for fatty acid composition.

Body weight, backfat thickness, and hams weight were recorded at slaughter. The backfat thickness was measured in two locations, coinciding with the sampling points (loin and ham), with a ruler.

### 4.2. Tissue Composition Analyses

The samples of subcutaneous adipose tissue were kept frozen (−80 °C) until their analysis. Tissue lipid extracts were obtained by Bligh and Dyer method [46]. The samples were made in duplicate. All the samples were processed in the same day, and their injection was done in less than a week, keeping the extracts at a temperature of −80 °C until their chromatographic injection. The precision of the fatty acid analyses measured as intraday coefficient of variability (CV) was less than 5%. Fat extracts were methylated in the presence of sulfuric acid and analyzed by gas chromatography. Previously fatty acid methyl ester (FAME) samples were identified by gas chromatography as described elsewhere [47], using an HP-6890 (Hewlett Packard, Avondale, PA, USA) gas chromatograph equipped with a flame ionization detector and a capillary column (HP-Innowax, 30 m by 0.32 mm i.d. and 0.25 μm polyethylene glycol-film thickness). A temperature program of 170 to 245 °C was used. The injector and detector were maintained at 250 °C. The carrier gas (helium) flow rate was 2 mL/min. For the identification of each fatty acid, standard patterns were used (Sigma, Alcobendas, Madrid, Spain). The concentration of individual fatty acids was calculated as % of total fatty acids, and the results were expressed as grams per 100 g of detected FAMEs. The response and corrections factors of each fatty acid are shown in Table S1.

Dietary FAs were extracted and quantified by the one-step procedure as described by Sukhija and Palmquist [48] in lyophilized samples. Pentadecanoic acid (C15:0) (Sigma, Alcobendas, Madrid, Spain) was used as internal standard. Previously methylated FA samples were identified by gas chromatography as described above. The results were expressed as grams per kg of feed.

### 4.3. Candidate Genes Expression Analyses by Quantitative PCR

The entire available adipose tissue sample (~50 to 100 mg) from each biopsy was used for total RNA extraction using the RiboPureTM RNA isolation kit (Ambion, Austin, TX, USA), following the manufacturer’s recommendations. The obtained RNA was quantified using a NanoDrop equipment (NanoDrop Technologies, Wilmington, DE, USA), and the RNA quality was assessed with an Agilent bioanalyzer device (Agilent Technologies, Palo Alto, CA, USA). The RNA integrity number values obtained for all the samples were in the range from 7.5 to 8.5. First-strand cDNA synthesis was carried out with Superscript II (Invitrogen Life Technologies, Paisley, UK) and random hexamers in a total volume of 20 μL containing 1 μg of total RNA, following the supplier’s instructions.

#### Selection of Candidate Genes

We selected thirteen candidate genes. Sterol regulatory element-binding transcription factor 1 (*SREBP1*) is recognized as the key transcription factor regulating lipogenic genes, Peroxisome proliferator activated receptor γ (*PPARG*) and Retinoic X receptor γ (*RXRG*) are involved in gene expression regulation of adipogenesis and lipogenesis [32,49]. Leptin (*LEP*) encodes a hormone produced by the adipose tissue, which controls energy balance and has local effects, inhibiting lipogenesis in the adipose tissue and promoting FA catabolism [50]. In addition, five candidate genes with a direct functional involvement in lipogenesis were selected: *ME1*, *SCD*, *FASN*, *ACACA*, and *ELOVL6*. Malic enzyme 1 (*ME1*) encodes a cytosolic, NADP-dependent enzyme that generates NADPH for fatty acid biosynthesis [51]. Stearoyl-CoA desaturase (*SCD*) encodes an endoplasmic reticulum enzyme that catalyzes the biosynthesis of monounsaturated fatty acids from saturated fatty acids [52]. Fatty acid synthase (*FASN*) encodes an important enzyme that catalyses the biosynthesis of saturated fatty acids (SFA), mainly palmitic acid [53]. Acetyl-CoA carboxylase alpha (*ACACA*) encodes an enzyme that catalyzes the carboxylation of acetyl-CoA to malonyl-CoA, a regulator of mitochondrial fatty-acid β-oxidation [54]. Fatty acid elongase 6 (*ELOVL6*) encodes an enzyme that catalyzes the rate-limiting step in the elongation cycle by controlling the fatty acid balance in mammals [55]. Moreover, four genes with a functional involvement in lipolysis, namely, *ATGL*, *HSL*, *G0S2*, and *PLIN1* were selected. Adipose triglyceride lipase (*ATGL*) and hormone-sensitive lipase (*HSL*) encode for two enzymes implicated in the complete hydrolysis of triacylglycerol (TAG) molecules in cellular lipid stores [56,57]. Under basal conditions, Perilipin 1 (*PLIN1*) restricts the access of cytosolic lipases (ATGL and HSL) to lipid droplets and thus promotes triacylglycerol storage. In times of energy deficit as fasting, PLIN1 is phosphorylated by protein kinase cAMP-dependent (PKA) and facilitates maximal lipolysis by ATGL and HSL [58]. The G0/G1 switch 2 (*G0S2*) gene is a negative regulator of lipolysis, whose activation is known to downregulate *ATGL* expression. Further studies showed that a 24 h fast downregulated the expression of *G0S2* and increased the expression of *ATGL* in the adipose tissue [59].

The expression the candidate genes was quantified by qPCR. Primer pairs were designed using Primer Select software (DNASTAR, Madison, WI, USA) from the available GENBANK and, or ENSEMBL sequences. Primer pairs covered different exons to assure the amplification of the cDNA. Information on primer sequences, efficiency, and amplicon lengths are indicated in Table 6.

Standard PCRs on cDNA were carried out to verify the amplicon sizes. Transcript quantification was performed using SYBR Green mix (Roche, Basel, Switzerland) in a LightCycler480 (Roche). The qPCR reactions were prepared in a total volume of 20 μL containing 2.5 μL of cDNA (1/20 dilution), 10 μL of SYBR Green mix, and 0.15 μM of both forward and reverse primers. As negative controls, mixes without cDNA were used. Cycling conditions were 95 °C for 10 min, followed by 45 cycles of 95 °C (15 s) and 60 °C (1 min), where the fluorescence was acquired. Finally, a dissociation curve to test PCR specificity was generated by one cycle at 95 °C (15 s) followed by 60 °C (20 s), and a ramp-up to 95 °C with acquired fluorescence during the ramp to 0.01 °C/s. Data were analyzed with the LyghtCycler480 software, release 1.5.0 SP4 (Roche). All points and samples were run in triplets as technical replicates, and dissociation curves were obtained for each individual replicate. Single peaks in the dissociation curves confirmed the specific amplification of the genes. For each gene, the PCR efficiency was estimated by standard curve calculation using four points of cDNA serial dilutions. The cycles to threshold (*Cp*) values were employed for the statistical analyses of differential expression. Data normalization was carried out by selecting the most stable endogenous genes out of *GAPDH*, *ACTB*, *TBP*, *18S*, *PPIA*, and *B2M*. The stability of the endogenous genes was tested with the Genorm software [60] and the Normfinder software [61]. The *ACTB* and *PPIA* genes were employed for normalization.

### 4.4. Statistical Analyses of Tissue Composition and Candidate Genes Expression

The influence of breed and diet on the fatty acid composition of carcass adipose tissue was analyzed with a linear model fitting breed, diet and the diet × breed interaction as fixed effects, and litter and box as random effects. The influence of breed and diet on FA composition was separately analyzed for each FA, or FA index in each tissue and layer. All the analyses were performed using MIXED procedure of SAS 9.1 (SAS Inst. Inc., Cary, NC, USA). Preliminary analyses were performed by fitting the fat layer (outer and inner) and the interaction fat layer × diet in each breed.

The method proposed by Steibel et al. [62] was employed for the statistical analysis of the gene expression data. This procedure simultaneously analyses the *Cp* values for the target and endogenous genes using a linear mixed model. The following model was used in a joint analysis of all the gene expression measures:

$$y_{gijklr}=TG_{gi}+P_{gj}+B_{gk}+A_{gl}+D_{ijkl}+e_{gijklr}$$

where $y_{gijklr}=-log_{2}\left(E_{g}^{-Cp_{gijklr}}\right)$, *E_g_* brings the PCR efficiency for each gene, *Cp_gijklr_* is the value obtained from the thermocycler software for the *g*th gene from the *r*th well in the *j*th qPCR plate and *k*th box, corresponding to the *l*th animal subjected to the *i*th treatment, *TG_gi_* is the specific effect of the *i*th treatment on the expression of gene g, *P_gj_*, *B_gk_* , and *A_gl_* are specific random effects on the expression in the *j*th qPCR plate of gene *g*, the *k*th box, and the *l*th pig, *D_ijkl_* is a random sample-specific effect common to all genes, and *e_gijklr_* is a residual effect.

Eight different groups were fitted to the model: dietary effects (two levels: HO and CH), two breeds (Iberian and Duroc pigs), and the four combinations of the two diets and fasting and feeding status in the Iberian and Duroc pigs. To test differences or interactions between classes in the expression rate of the genes of interest (*diff_TG_*), different contrasts were performed between the appropriate estimates of *TG* levels. The significance of *diff_TG_* estimates was determined with the *t* statistic. The adjusted *p*-values were calculated using the correction method of Benjamini and Hochberg [63] for accounting the multiplicity of comparisons, with the Cheverud–Nyholt formula for the number of effective tests of Moskina and Schmidt [64]. The adjusted *p*-values for all analyses are indicated in the tables of results. Nevertheless, in order to perform a wide interpretation of the results, taking into account not only the significance of each individual analysis, but also the profiles and concordance of the gene expression results for the genes grouped by functional category, the results with nominal *p*-values < 0.05 were considered within the discussion. For this purpose, the multiple test correction may be considered too demanding.

To obtain fold change values (*FC*) from the estimated *diff_TG_* values, the following equation was applied: $FC=2^{-diff_{TG}}$. The standard errors of fold change values (*SE* (*FC*)) were calculated from the standard error of the estimated differences (*SE*), using a similar transformation: *SE* (*FC*) = 2^−*SE*^. The asymmetric confidence intervals (95% CI) were calculated for each FC value by using the *SE* values: 95% CI from $2^{-\left(diff_{TG}+t\left(\gamma ,\;0.975\right)\cdot SE\right)}$ to $2^{-\left(diff_{TG}-t\left(\gamma ,\;0.975\right)\cdot SE\right)}$, where *t* (γ,0.975) is the 97.5 quantile of the Student-*t* distribution with γ degrees of freedom. In our analyses, γ ranged from 6 to 133.

## 5. Conclusions

The results of the present work provide a relevant phenotypic and transcriptional characterization of lipid metabolism processes in the adipose tissue of pure Iberian and Duroc growing pigs, bred and managed in identical conditions. Our findings are of high scientific value and agree with the high lipogenic and desaturation potential, thrifty genotype, and leptin resistance of the Iberian pig breed, and support the persistence of de novo lipogenesis during fasting in the obese Iberian pigs. A medium-long period of fasting is needed in this breed to induce an adaptation in the function of lipogenic genes. Joint results support the usefulness of the Iberian pig as animal model for obesity and metabolic disorders

On the other hand, the regulation of lipolytic genes in the adipose tissue by breed, status, and diet factors, scarcely studied to date, is much more complex and subjected to intricate interactions. The effects of the FA profile of the diet on lipid metabolism are small and conditional on other factors. The joint results deepen the knowledge of the molecular basis and regulation processes of lipid metabolism in both pig breeds and highlight the complexity of this regulation, especially in relation to lipolysis processes.

The phenotipic characterization of the experimental groups provides novel and relevant information regarding the usefulness of supplemented diets to improve the pork quality, in both the Iberian and Duroc pig breeds, with practical implications.

## Figures and Tables

**Table 1:** Least-squares means and standard errors (SEM) of fatty acid percentages in subcutaneous backfat (inner layer) from Iberian and Duroc pigs fed with a diet enriched with high-oleic sunflower oil (HO) or a standard diet with carbohydrates as energy source (CH).

Fatty Acid ^1^	Diet CH (*n* = 22)		Diet HO (*n* = 27)		*p*-Value	Adjusted *p*-Value	DUROC (*n* = 19)		IBERIAN (*n* = 30)		*p*-Value	Adjusted *p*-Value
	MEAN	SEM	MEAN	SEM			MEAN	SEM	MEAN	SEM		
C14:0	1.24	0.03	1.12	0.03	0.05	ns	1.08	0.03	1.25	0.03	0.005	ns
C16:0	23.68	0.23	20.91	0.23	<0.0001	0.0007	20.32	0.27	24.27	0.20	<0.0001	0.0007
C16:1n-9	0.33	0.01	0.44	0.01	<0.0001	0.0007	0.45	0.02	0.31	0.01	<0.0001	0.0007
C16:1n-7	1.97	0.09	1.60	0.08	0.009	ns	1.97	0.08	1.60	0.10	0.01	ns
C17:0	0.46	0.03	0.39	0.03	0.14	ns	0.41	0.04	0.44	0.03	0.60	ns
C17:1	0.35	0.02	0.28	0.02	0.001	0.04	0.29	0.02	0.34	0.02	0.15	ns
C18:0	13.35	0.24	10.35	0.22	<0.0001	0.0007	10.31	0.26	13.39	0.21	<0.0001	0.0007
C18:1n-9	40.23	0.53	46.49	0.50	<0.0001	0.0007	44.20	0.61	42.53	0.46	0.06	ns
C18:1n-7	1.64	0.13	1.68	0.12	0.78	ns	1.77	0.15	1.55	0.12	0.30	ns
C18:2n-6	13.42	0.22	13.41	0.20	0.99	ns	16.13	0.25	10.70	0.20	<0.0001	0.0007
C18:3n-3	0.84	0.01	0.88	0.01	0.02	ns	1.00	0.02	0.72	0.01	<0.0001	0.0007
C18:4n-3	0.06	0.00	0.07	0.00	0.0004	0.03	0.07	0.00	0.06	0.00	0.01	ns
C20:0	0.22	0.01	0.18	0.01	0.002	ns	0.18	0.01	0.22	0.01	0.01	ns
C20:1n-9	0.97	0.03	1.03	0.02	0.03	ns	0.90	0.03	1.10	0.03	0.0002	0.01
C20:2	0.68	0.01	0.61	0.01	0.004	ns	0.71	0.02	0.59	0.01	0.0002	0.01
C20:4n-6	0.22	0.01	0.22	0.01	0.72	ns	0.26	0.01	0.18	0.01	<0.0001	0.0007
C20:3n-3	0.12	0.00	0.11	0.00	0.02	ns	0.12	0.00	0.11	0.00	0.01	ns
C22:4n-6	0.10	0.00	0.09	0.00	0.27	ns	0.10	0.01	0.09	0.00	0.25	ns
C22:5n-3	0.09	0.02	0.09	0.01	0.69	ns	0.07	0.02	0.11	0.01	0.11	ns
C22:6n-3	0.03	0.01	0.05	0.01	0.20	ns	0.04	0.01	0.04	0.01	0.98	ns
SFA ^2^	38.93	0.55	32.96	0.51	<0.0001	0.0007	32.31	0.61	39.57	0.47	<0.0001	0.0007
MUFA ^3^	45.13	0.79	51.24	0.73	<0.0001	0.0007	49.28	0.89	47.09	0.72	<0.0001	0.0007
PUFA ^4^	14.86	0.29	14.93	0.26	0.34	ns	17.79	0.32	12.01	0.26	<0.0001	0.0007
n-6/n-3	12.0	0.01	11.43	0.01	0.04	ns	12.78	0.01	11.19	0.01	<0.0001	0.0007

^1^ Fatty acid composition is expressed as percentage (*wt*/*wt*) of total fatty acids. ^2^ SFA, ^3^ MUFA, ^4^ PUFA = sum of saturated, monounsaturated and polyunsaturated fatty acids, respectively, ns: not significant.

**Table 2:** Least-squares means and standard errors (SEM) of fatty acid percentages in subcutaneous ham fat (inner layer) from Iberian and Duroc pigs fed with a diet enriched with high-oleic sunflower oil (HO) or a standard diet with carbohydrates as energy source (CH).

Fatty Acid ^1^	Diet CH (*n* = 22)		Diet HO (*n* = 27)		*p*-Value	Adjusted *p*-Value	DUROC (*n* = 19)		IBERIAN (*n* = 30)		*p*-Value	Adjusted *p*-Value
	MEAN	SEM	MEAN	SEM			MEAN	SEM	MEAN	SEM		
C14:0	1.24	0.02	1.18	0.02	0.02	ns	1.15	0.02	1.27	0.02	0.0008	0.05
C16:0	22.57	0.22	20.54	0.20	<0.0001	0.0007	20.38	0.23	22.73	0.18	<0.0001	0.0007
C16:1n-9	0.28	0.01	0.39	0.01	<0.0001	0.0007	0.36	0.02	0.31	0.01	0.02	ns
C16:1n-7	2.57	0.08	2.16	0.07	<0.0001	0.0007	2.28	0.09	2.45	0.07	0.17	ns
C17:0	0.33	0.03	0.41	0.03	0.17	ns	0.33	0.04	0.42	0.03	0.13	ns
C17:1	0.40	0.02	0.31	0.02	0.0009	0.06	0.30	0.03	0.41	0.02	0.0099	ns
C18:0	10.48	0.21	8.79	0.19	<0.0001	0.0007	9.33	0.23	9.94	0.18	0.05	ns
C18:1n-9	44.75	0.51	49.40	0.48	<0.0001	0.0007	47.26	0.57	46.89	0.43	0.64	ns
C18:1n-7	1.84	0.12	1.69	0.11	0.27	ns	1.67	0.13	1.85	0.10	0.34	ns
C18:2n-6	12.11	0.19	11.87	0.17	0.53	ns	13.58	0.21	10.41	0.16	<0.0001	0.0007
C18:3n-3	0.80	0.02	0.80	0.01	0.45	ns	0.87	0.02	0.73	0.02	<0.0001	0.0007
C18:4n-3	0.08	0.00	0.09	0.00	0.0016	ns	0.09	0.00	0.08	0.00	0.26	ns
C20:0	0.16	0.01	0.14	0.00	<0.0001	0.0007	0.15	0.01	0.15	0.01	0.50	ns
C20:1n-9	1.00	0.02	1.04	0.02	0.05	ns	0.91	0.02	1.13	0.02	<0.0001	0.0007
C20:2	0.67	0.01	0.61	0.01	0.0004	0.03	0.68	0.01	0.61	0.01	<0.0001	0.0007
C20:4n-6	0.25	0.01	0.25	0.01	0.56	ns	0.27	0.01	0.23	0.01	0.04	ns
C20:3n-3	0.13	0.00	0.13	0.00	0.03	ns	0.13	0.00	0.11	0.00	0.02	ns
C22:4n-6	0.10	0.00	0.09	0.00	0.05	ns	0.10	0.00	0.10	0.00	0.46	ns
C22:5n-3	0.13	0.01	0.11	0.01	0.30	ns	0.09	0.02	0.15	0.01	0.03	ns
C22:6n-3	0.07	0.01	0.07	0.01	0.43	ns	0.07	0.01	0.07	0.01	0.78	ns
SFA ^2^	34.78	0.48	31.06	0.44	<0.0001	0.0007	31.33	0.52	34.52	0.42	<0.0001	0.0007
MUFA ^3^	50.43	0.75	54.68	0.70	<0.0001	0.0007	52.48	0.83	52.63	0.64	0.99	ns
PUFA ^4^	13.67	0.25	13.41	0.23	0.34	ns	15.20	0.28	11.87	0.22	<0.0001	0.0007
n-6/n-3	11.56	0.01	10.19	0.01	0.04	ns	11.16	0.01	9.42	0.01	<0.0001	0.0007

^1^ Fatty acid composition is expressed as percentage (*wt*/*wt*) of total fatty acids. ^2^ SFA, ^3^ MUFA, ^4^ PUFA= sum of saturated, monounsaturated and polyunsaturated fatty acids, respectively, ns: not significant.

**Table 3:** Main effects of breed (Iberian/Duroc), feeding/fasting status and diet (HO/CH) on expression of candidate genes in ham adipose tissue.

Gene	Breed (Iberian/Duroc) (*n* = 30/19)				Status (Feeding/Fasting) (*n* = 48/48)				Diet (HO ^1^/CH ^2^) (*n* = 27/22)			
	Fold Change	95% CI ^3^	*p*-Value	Adjusted *p-*Value	Fold Change	95% CI ^3^	*p*-Value	Adjusted *p-*Value	Fold Change	95% CI ^3^	*p*-Value	Adjusted *p-*Value
*RXRG*	1.33	0.90–1.96	0.1424	0.196	1.27	1.08–1.49	0.0051	0.003	1.12	0.91–1.39	0.2720	0.913
*PPARG*	1.07	0.84–1.36	0.5434	0.213	1.22	1.07–1.38	0.0029	0.011	0.87	0.69–1.09	0.168	0.328
*SREBP1*	0.87	0.65–1.17	0.3153	0.434	1.11	0.93–1.32	0.234	0.322	0.99	0.74–1.33	0.964	0.970
*LEP*	2.85	1.23–6.64	0.0174	0.075	2.05	1.69–2.48	<.0001	0.001	0.99	0.68–1.45	0.970	0.970
*ME1*	1.80	1.03–3.14	0.0427	0.024	1.12	1.00–1.24	0.0470	0.117	1.18	0.70–2.00	0.4912	0.201
*SCD*	2.21	1.49–3.27	0.0009	0.003	1.30	1.21–1.40	<.0001	0.001	1.05	0.74–1.49	0.7304	0.893
*ACACA*	1.49	0.90–2.46	0.1068	0.147	2.10	1.87–2.35	<.0001	0.001	0.97	0.61–1.53	0.8709	0.871
*FASN*	1.36	0.95–1.94	0.0820	0.113	1.13	1.02–1.24	0.0196	0.036	1.16	0.81–1.63	0.3655	0.804
*ELOVL6*	1.60	1.06–2.40	0.0280	0.039	1.22	1.10–1.37	0.0005	0.001	0.94	0.68–1.31	0.664	0.809
*ATGL*	0.89	0.78–1.01	0.0640	0.088	0.99	0.92–1.07	0.811	0.811	1.01	0.92–1.11	0.799	0.879
*HSL*	0.94	0.79–1.13	0.4870	0.670	0.96	0.89–1.03	0.275	0.336	0.96	0.82–1.14	0.640	0.805
*PLIN1*	1.93	0.90–4.14	0.0873	0.120	1.07	0.92–1.25	0.399	0.439	0.79	0.63–1.00	0.050	0.046
*G0S2*	0.89	0.47–1.70	0.6887	0.814	1.50	1.16–1.95	0.003	0.007	0.82	0.51–1.30	0.321	0.501

^1^ HO = high oleic diet with high oleic sunflower oil; ^2^ CH = carbohydrate diet without added fat; ^3^ CI: Confidence Interval.

**Table 4:** Breed × status, breed × diet and diet × status interaction effects on candidate genes expression.

Gene	Breed × Status	Breed × Diet	Diet × Status
	Nominal *p*-Value	Nominal *p*-Value	Nominal *p*-Value
*RXRG*	ns	ns	ns
*PPARG*	ns	ns	ns
*SREBP1*	ns	ns	0.03
*LEP*	ns	ns	ns
*ME1*	0.07	0.07	ns
*SCD*	0.03	ns	ns
*ACACA*	0.0001 *	ns	0.004 *
*FASN*	ns	ns	ns
*ELOVL6*	ns	ns	ns
*ATGL*	ns	0.006	0.02
*HSL*	ns	0.07	0.03
*PLIN1*	ns	0.07	0.0004 *
*G0S2*	ns	ns	ns

* Significant adjusted *p*-values; ns: not significant.

**Table 5:** Calculated analysis ^1^ and fatty acid composition of the experimental diets (g/kg, as-fed basis).

Diet	Carbohydrate (CH) ^2^	High Oleic (HO) ^3^
Chemical composition, g/kg of feed		
Moisture	87.4	88.81
Lipids	24.53	77.65
Crude protein	156	156
Crude fiber	29.71	45.27
Nitrogen-free Extractives	515.75	404.39
Ash	44.34	67.91
Main Fatty acids, g/kg of feed		
C14:0	0.14	0.13
C16:0	4.83	7.19
C18:0	0.84	1.83
C18:1n-9	9.47	36.82
C18:2n-6	14.24	16.68
C18:3n-3	0.99	1.21

^1^ According to Fundación Española Desarrollo Nutrición Animal (2010); ^2^ CH = Carbohydrate diet without added fat; ^3^ HO = High oleic diet with high oleic sunflower oil.

**Table 6:** Primer design for qPCR and PCR efficiencies.

Gene Symbol	Gene Name	GenBank ID	Forward Primer Sequence	Reverse Primer Sequence	Efficency (%)
*RXRG*	Retinoic X receptor gamma	DQ866834.1	GGGGTTGGCTCCATCTTTGA	ACCTGCCCGGCTGTTCTG	84.8
*PPARG*	Peroxisome proliferator activated gamma	NM_214379.1	GGCGAGGGCGATCTTGACAG	GATGCGAATGGCCACCTCTTT	93.5
*SREBP1*	Sterol regulatory element binding transcription factor 1	NM_214157.1	AGTTGAGCCCTGCCCCCGTGTTG	CTGCTGGATCTGCGAGGTCA	91.5
*LEP*	*Leptin*	NM_213840.1	GGCCCTATCTGTCCTACGTTGAAG	TGGAAGGCAGACTGGTGAGGAT	92.8
*ME1*	Malic enzyme	XM_001924333.4	GCCGGCTTTATCCTCCTCT	TCAAGTTTGGTCTGTATTTTCTGG	86.5
*SCD*	Stearoyl- CoA desaturase	NM_213781.1	TCCCGACGTGGCTTTTTCTTCTC	CTTCACCCCAGCAATACCAG	89.6
*ACACA*	Acetyl-CoA carboxylase alpha	NM_001114269.1	CTGAGAGCTCGTTTTGAAGGAATA	TTTACTAGGTGCAAGCCAGACAT	85.2
*FASN*	Fatty acid synthase	NM_001099930.1	GCAGGCGCGTGATGGGAATGGTG	GCCCGAGCCCGAGTGGATGAGCA	85.1
*ELOVL6*	Fatty acid elongase 6	XM_013978957.1	AGAACACGTAGCGACTCCGAAGAT	GACATGCCGACCGCCAAAGATAA	89.5
*ATGL*	Adipose triglyceride lipase	NM_001098605.1	GCACCTTCATTCCCGTGTAC	TTGTCTGAGATGCCACCGTC	87.2
*HSL*	Hormone sensitive lipase	NM_214315.1	CCCCCGTGCGCTGGAGGAGT	GGGAGGGGGAGGCGGCAGAC	82.8
*PLIN1*	Perilipin 1	NM_001038638.1	CCCCCTGGTGGCGTCTGTAT	ACTGGAGGGCCGGTATCTTTTCT	87.9
*G0S2*	G0/G1 switch 2	NM_001286804	GAGAGCCCGGAGCCGAGATGGA	CCGAGCACCGCGCCGAGAAA	83.7
*ACTB*	Beta-actin	XM_003124280.4	TCTGGCACCACACCTTCT	TGATCTGGGTCATCTTCTCAC	90.7
*PPIA*	Peptidylprolyl isomerase A	NM_214353	GGGAGAAAGGATTTGGTTAT	ATGGACAAGATGCCAGGAC	96.9

## Supplementary Materials

Supplementary materials can be found at www.mdpi.com/1422-0067/19/1/22/s1.

## Abbreviations

HO	High Oleic diet
CH	Carbohydrates diet
FA	Fatty acid
SFA	Saturated fatty acid
MUFA	Monounsaturated fatty acid
PUFA	Polyunsaturated fatty acid
FC	Fold-Change
TAG	Triacylglycerols
PKA	Protein kinase cAMP-dependent
