# Supplementary material for: Modulatory Effects of Breed, Feeding Status, and Diet on Adipogenic, Lipogenic, and Lipolytic Gene Expression in Growing Iberian and Duroc Pigs
Source: Int J Mol Sci. 2017 Dec 22;19(1):22. doi: 10.3390/ijms19010022 (PMC5795973; doi:10.3390/ijms19010022)
Supplement: Supplementary file 1 [file ijms-19-00022-s001.pdf]

supplementary figure 1: Breed*diet interactions

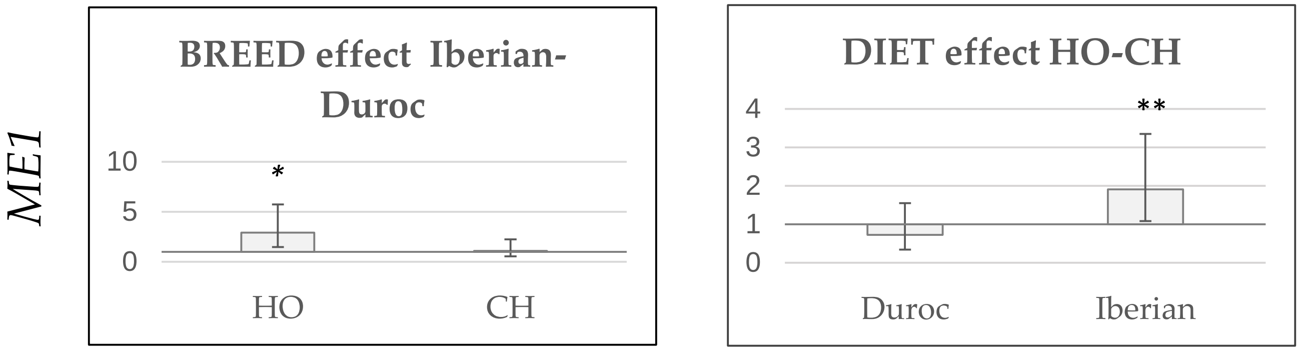

Breed*diet interaction *P*<0.07

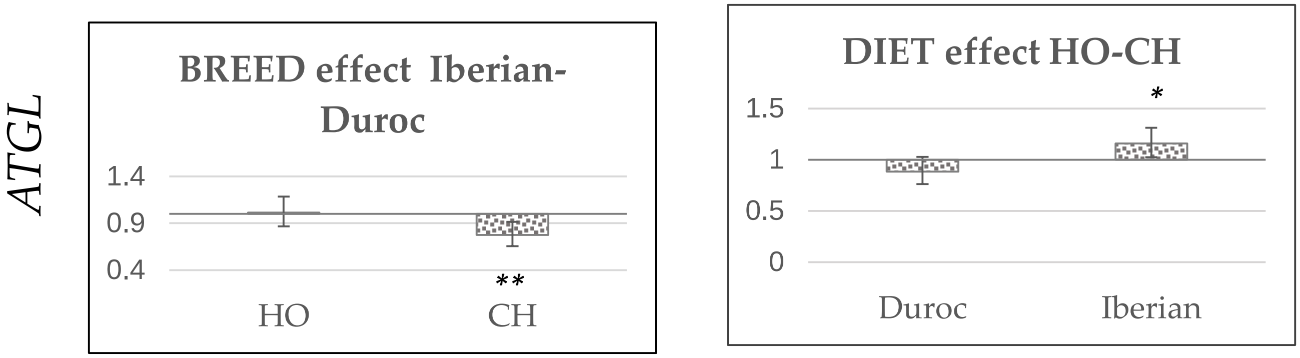

Breed*diet interaction *P*<0.0006

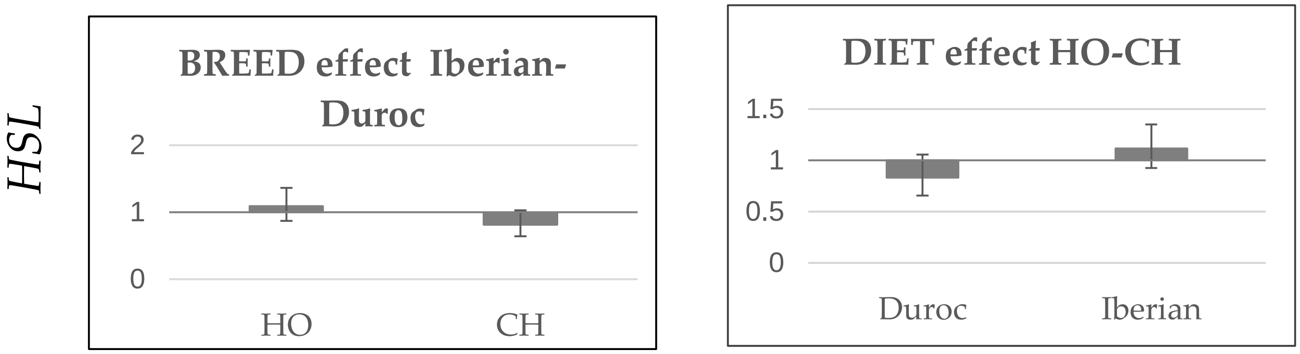

Breed*diet interaction *P*<0.07

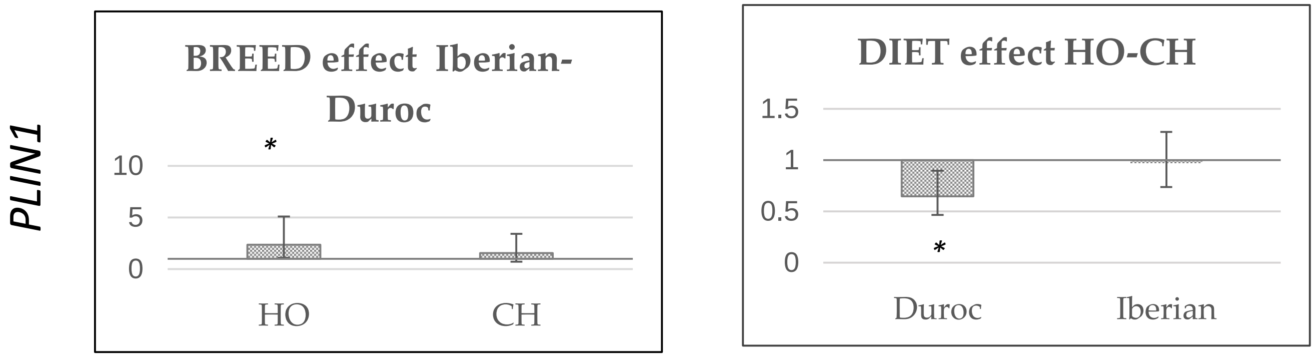

Breed*diet interaction *P*<0.08

supplementary figure 2: Diet*status interactions

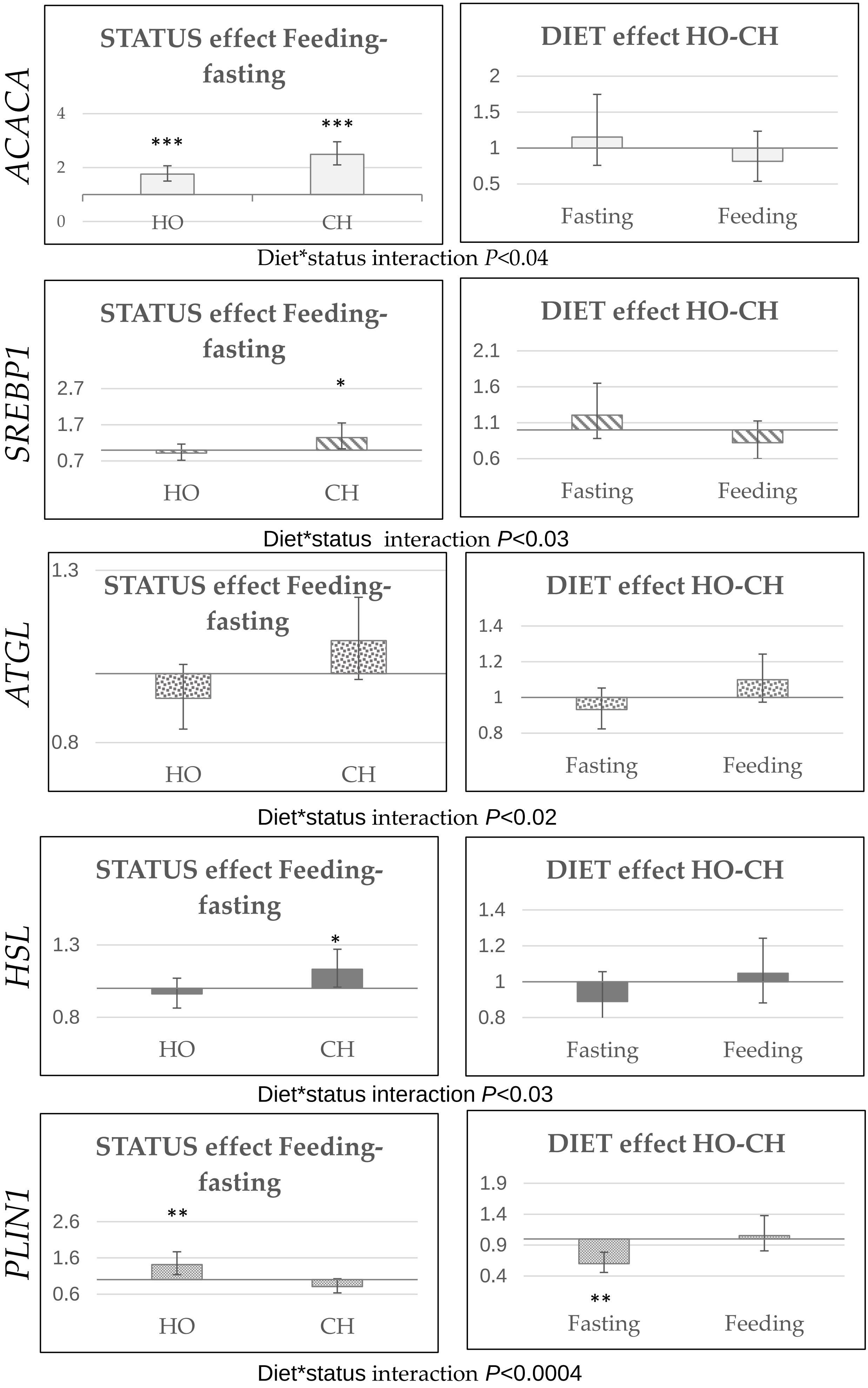

Table S1. Response Factors and Corrections Factors of Fatty acids.

	Molecular Mass (Mx)	Number of Carbon atoms (nx-1)	Response Factor FID* (Fx)	Correction Factor FID* (fx)
C14:0	242.38	14	1.44	1.02
C16:0	270.42	16	1.41	1.00
C16:1 n-7	268.41	16	1.40	0.99
C16:1 n-9	268.41	16	1.40	0.99
C17:0	284.45	17	1.39	0.99
C17:1	282.44	17	1.38	0.98
C18:0	298.47	18	1.38	0.98
C18:1 n-9	296.46	18	1.37	0.97
C18:1 n-7	296.46	18	1.37	0.97
C18.2 n-6	294.45	18	1.36	0.97
C18:3 n-3	292.43	18	1.35	0.96
C18:4 n-3	290.42	18	1.34	0.95
C20:0	326.52	20	1.36	0.97
C22:1 n-9	324.51	20	1.35	0.96
C20:2	322.50	20	1.34	0.95
C20:3	320.48	20	1.33	0.95
C20:4	318.47	20	1.33	0.94
C22:5 n-3	344.50	22	1.30	0.93
C22:6 n-3	342.49	22	1.30	0.92

* FID: Flame Ionization Detector
